# Tetracycline and Sulfonamide Antibiotic Resistance Genes in Soils From Nebraska Organic Farming Operations

**DOI:** 10.3389/fmicb.2018.01283

**Published:** 2018-06-28

**Authors:** Marlynn Cadena, Lisa M. Durso, Daniel N. Miller, Heidi M. Waldrip, B. L. Castleberry, Rhae A. Drijber, Charles Wortmann

**Affiliations:** ^1^Department of Biological Sciences, College of Science, The University of Texas at El Paso, El Paso, TX, United States; ^2^Agroecosystem Management Research Unit, Agricultural Research Service, United States Department of Agriculture, Lincoln, NE, United States; ^3^Conservation and Production Research Laboratory, Agricultural Research Service, United States Department of Agriculture, Bushland, TX, United States; ^4^Department of Agronomy and Horticulture, University of Nebraska–Lincoln, Lincoln, NE, United States

**Keywords:** soil, antibiotic resistance, antibiotic resistance gene, ARG, organic, farm, agriculture, environment

## Abstract

There is widespread agreement that agricultural antibiotic resistance should be reduced, however, it is unclear from the available literature what an appropriate target for reduction would be. Organic farms provide a unique opportunity to disentangle questions of agricultural antibiotic drug use from questions of antibiotic resistance in the soil. In this study, soil was collected from 12 certified organic farms in Nebraska, evaluated for the presence of tetracycline and sulfonamide resistance genes (*n* = 15 targets), and correlated to soil physical, chemical, and biological parameters. Tetracycline and sulfonamide antibiotic resistance genes (ARGs) were found in soils from all 12 farms, and 182 of the 196 soil samples (93%). The most frequently detected gene was tetG (55% of samples), followed by *tet*(Q) (49%), *tet*(S) (46%), *tet*(X) (30%), and *tetA*(P) (29%). Soil was collected from two depths. No differences in ARGs were observed based on soil depth. Positive correlations were noted between ARG presence and soil electrical conductivity, and concentrations of Ca, Na, and Mehlich-3 phosphorus. Data from this study point to possible relationships between selected soil properties and individual tetracycline resistance genes, including *tet*(O) which is a common target for environmental samples. We compared organic farm results to previously published data from prairie soils and found significant differences in detection frequency for 12 genes, eight of which were more commonly detected in prairie soils. Of interest, when tetracycline ARG results were sorted by gene mechanism, the efflux genes were generally present in higher frequency in the prairie soils, while the ribosomal protection and enzymatic genes were more frequently detected in organic farm soils, suggesting a possible ecological role for specific tetracycline resistance mechanisms. By comparing soil from organic farms with prairie soils, we can start to determine baseline effects of low-chemical input agricultural production practices on multiple measures of resistance.

## Introduction

The global emergence of antibiotic resistance has led to the immediate need to find ways to mitigate resistance in the environment. Agricultural antibiotic resistance is an issue that has gained national and international attention (Topp et al., 2017), and there is concern that resistance from cropland and livestock will be transferred through the environment and cause untreatable infectious disease in people and animals (Durso and Cook, 2014). In conventional food animal production, livestock, and poultry are commonly given antibiotics to treat and prevent illnesses (Du and Liu, 2012). However, research has indicated that only 10 to 20% of antibiotics administered are absorbed into animal tissue: the majority is excreted in manure (Ok et al., 2011). The presence of antibiotic residues in manure may lead to selection and proliferation of strains of antibiotic resistant bacteria (ARB); thus, manure from livestock facilities is a source of antibiotic drugs, ARB, and antibiotic resistance genes (ARGs) excreted into the environment (Binh et al., 2008; Heuer et al., 2011). There is widespread agreement and support for the idea that agricultural antibiotic resistance should be reduced, with an emphasis on reducing transfer of resistance from practices such as land application of animal manures (Heuer et al., 2011; Pruden et al., 2013; Marti et al., 2014), and spraying of antibiotics to control bacterial disease in fruit crops (Stockwell and Duffy, 2012). However, the details of a realistic reduction target are elusive. In order to develop effective methods to reduce resistance, it is important to first obtain baseline information on how basic agricultural practices are involved in resistance transfer. There remain many knowledge gaps surrounding the basic ecology of antibiotic resistance on farms and in fields, such as how variable is any particular measure of resistance within or between farms? And from a human and animal health standpoint, which types of resistance should be measured or tracked?

Organic farms provide a unique and valuable opportunity to disentangle questions of agricultural drug use from questions of antibiotic resistance. Since antibiotic drugs use is severely restricted in organic operations, these farms provide a natural starting place for assessing background and baseline levels of ARB and ARG in agricultural production settings (Rothrock et al., 2016b).

In Nebraska, over 90% of the land mass is devoted to agriculture, with cattle, corn, soybeans, hogs, and eggs being the top agricultural commodities, in order of value (Nebraska Department of Agriculture [NDA], 2017). In 2016, the USDA National Agricultural Statistics Service reported a total of 48,400 farm operations in Nebraska (National Agriculture Statistics Service [NASS], 2016). Of these, 267 were certified organic according to the USDA Agricultural Marketing Service’s Organic Integrity Database (United States Department of Agriculture [USDA], 2017). A previous study characterized ARB/ARG in native Nebraskan prairie soils, providing a reference point for resistance in soils with minimal anthropogenic inputs (Durso et al., 2016); however, data on resistance in organic farm soils from this region are lacking. Here we assess prevalence and distribution of selected tetracycline and sulfonamide resistance genes in soil from 12 USDA certified organic farming operations in Nebraska. Resistance gene distributions were compared within and among different organic operations and at different soil sampling depths. In addition, this study explored relationships between ARGs and soil physical, chemical, and biological characteristics. There is some indication that soil nutrient levels may impact the prevalence of ARB/ARG (Udikovic-Kolic et al., 2014; Zhou et al., 2017); therefore, we hypothesize that relationships will be observed between ARG frequency and selected soil characteristics.

## Materials and Methods

### Soil Collection and Analyses

Soil samples were collected from 12 certified organic farms in Nebraska. The crops grown are listed in **Table 1**. There were no animals on pasture at the time of collection. Information on whether or not manure had been used as a soil amendment within the last three years is provided in **Table 1**. A total of 98 soil cores (15.24 cm) were collected between May 22 and June 6, 2013. Aboveground residue and large roots were removed. Soil for microbiological analysis was collected using a gardener’s trough which was cleaned following each sample, placed in polyethylene bags and immediately stored on ice for transport to the laboratory. Soil for chemical analysis and aggregate stability were collected using a spade. In total, 98 cores were collected from 12 farms. Samples were collected at two depths (0.0–7.6 cm and 7.6–15.2 cm), homogenized by hand-mixing of the bag, and stored at -80°C, resulting in a total of 196 soil samples that were evaluated for ARG targets. Soil analyses, including determination of coarse particular organic matter (CPOM), fine particulate organic matter (FPOM), microaggregates (MicAg), large and small macroaggregates (Lmac, Smac), pH, electrical conductivity (EC), and fatty acid profiles were performed as part of a separate study, using methods that have previously been described (Cambardella and Elliott, 1994; Drijber et al., 2000; Cambardella et al., 2001; Grigera et al., 2006). Chemical analyses were performed at Ward Laboratories, Kearney, NE, United States. Briefly, Nitrate-nitrogen was extracted using a Ca solution to flocculate soil clays, and analyzed using a cadmium reduction procedure, with a flow injection analyzer; phosphorus was extracted by the Mehlich P-3 test, using an extracting solution of 0.013 N HNO_3_ and 0.015 N NH_4_F; potassium was extracted using 1 N ammonium acetate, and analyzed with a flame emission mode of an atomic absorption spectrophotometer; sulfur was extracted using calcium phosphate, followed by barium sulfate turbidity determined by flow injection analysis; micronutrients were extracted with a chelated DTPA solution and Ca and Mg were extracted using an ammonium acetate solution an measured with an atomic absorption spectrophotometer.

**Table 1:** Description of sample collection sites.

Farm	No. of cores	Crop at time of collection	Previous crop(s)	Recent manure
1	6	W. wheat	Soybeans, corn	Yes
2	4	Warm and cool perennial grasses	Warm and cool perennial grasses	Yes
3	6	Wheat, fallow, millet	Wheat, fallow, millet	Yes
4	2	Mix vegetables	Mix vegetables	No
5	11	Oats, corn, alfalfa, pasture mix^∗^	Oats, corn, alfalfa, pasture mix^∗^	Yes
6	7	Pasture, oats, w. wheat	Soy, pasture, oats, corn, w. wheat	Yes
7	10	Corn	Soybeans, corn	Yes
8	10	Soy, oats, alfalfa, corn, pasture	Soy, oats, alfalfa, corn, pasture	Yes
9	7	Pasture, oats, corn, sorghum, millet	Pasture, oats, corn, sorghum, millet	Yes
10	8	Soybeans	Corn	Yes
11	16	Popcorn, hay, pasture, soy, barley	Popcorn, hay, pasture, soy, barley	Yes
12	11	Wheat, soy, corn, alfalfa, pasture, oats	Wheat, soy, corn, alfalfa, pasture, oats	Yes

*Samples for each depth were formed in the field at the time of sampling, resulting in two soil samples location. A “yes” in the “recent manure” column means that some or all of the samples from that farm were collected from areas which had received manure within the last three years. Not all fields within a farm necessarily had “recent manure.” ^∗^Pasture mix contains (pasture, oats, buck wheat, turnip, radish). “W. wheat” indicates winter wheat. “Soy” indicates soybean.*

### Molecular Analyses

Isolation and purification of DNA from bulk soil samples (*n* = 196) was conducted with the DNeasy PowerSoil Kit (Qiagen Sciences Inc., Germantown, MD, United States) according to the manufacturer’s protocol. A Bead Ruptor 24 homogenizer (OMNI International, Kennesaw, GA, United States) was used for sample mixing during DNA isolation. Purified DNA was quantified using a NanoDrop3300 (ThermoFisher, Waltham, MA, United States), and used directly in the polymerase chain reactions (PCRs). All samples were subjected to the PCR for detection of 15 tetracycline and sulfonamide resistance genes (**Supplementary Table S1**), resulting in 2,940 total PCR assays performed. There are 29 genes known to code for resistance to tetracyclines (Roberts, 2005), and four genes known to code for resistance to sulfonamide (Razavi et al., 2017). We chose a subset of the tetracycline resistance genes for which multiplex PCR reactions had previously been described (Ng et al., 2001). Since *sul*1 is one of the most frequently detected sulfonamide resistance genes (Phuong Hoa et al., 2008), and since it has been closely associated with class 1 integrons responsible for transfer of ARGs between bacteria, we chose *sul*1 for this study. The PCR reactions were performed as previously described for ARG in soils (Ng et al., 2001; Pei et al., 2006; Durso et al., 2016). In brief, thermocycling conditions were one cycle of 94°C for 2 min, followed by 30 cycles of denaturation at 94°C (60 s), annealing at primer-specific temperatures (see **Supplementary Table S1**) for 60 s, and extension at 72°C (90 s), with a 5-min final extension at 72°C for 5 min. Bands were visualized using Invitrogen SYBR Safe DNA gel stain (Life Technologies, Carlsbad, CA, United States) added directly to tris-acetate-EDTA 2% agarose gels, and documented using a UVP Gel Doc-It^TS3^ imaging system (UVP, LLC, Upland, CA, United States). Note that standard PCR assays can only report the presence or absence of the selected target, and do not provide information on the amount of the targets in the sample.

### Data Analysis

The SAS GLM procedure was used to determine differences for each of the soil physical, chemical, and biological parameters between samples positive and negative for each ARG target (SAS Institute, 2008). Results are reported for both *P* ≤ 0.05 and *P* ≤ 0.1 probability levels. Significant correlations between number of positive ARG targets per sample and various soil parameters were identified at the (*P* ≤ 0.05) level using Pearson correlation coefficients. The MEANS procedure was used to examine farm-level depth-based differences in soil parameters. Differences in the proportions of ARG between surface and deeper cores or between organic farms and prairies were determined using the TABLES statement in PROC FREQ and designating the CHISQ option (equivalent to a Z test for the equality of proportions). Antibiotic fingerprinting was performed as previously described by concatenating individual ARG target results (Durso et al., 2011). Individual ARG assay results were coded as 1 if the target was detected in the sample and 0 if the target was not detected in the sample. Then, these results were combined into a 14-digit binary “fingerprint” for each sample, and used for comparison purposes.

## Results

**FIGURE 1:**
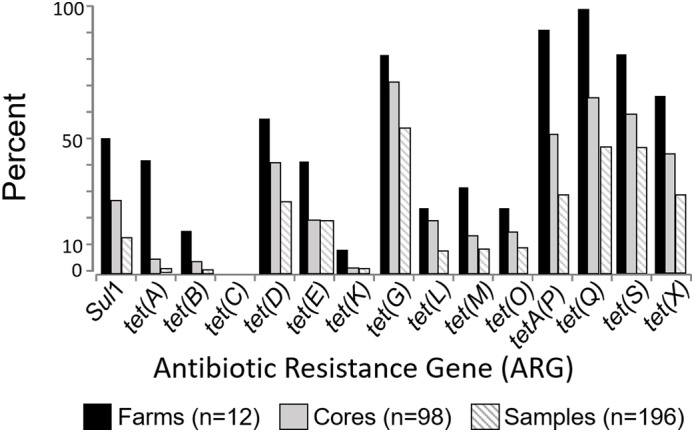
Farm, core, and sample level prevalence of selected antibiotic resistance genes.

Tetracycline and/or sulfonamide resistance genes were found in soils collected from all 12 organic farms (100%) (**Supplementary Figure S1**), in 94 of 98 cores (96%) and in 178 of the 196 soil samples (91%). This study examined 15 ARG targets, and all but one [*tet*(C)] were found in at least one of the 196 soil samples (**Figure 1**). The most frequently detected genes at the farm level (*n* = 12 farms) were *tet*(G), *tetA*(P), and *tet*(Q) with 83%, 92%, and 100% of the farms positive for each of these targets, respectively (**Figure 1**). At the individual soil sample level (*n* = 196 samples), the most frequently detected genes were *tet*(G) (55% of samples), followed by *tet*(Q) (49%), *tet*(S) (46%), *tet*(X) (30%), and *tetA*(P) (29%) (**Figure 1**). Most of the samples (91%) were positive for at least one of the 15 targets, and 82% were positive for two or more of the tested ARGs. The number of positive ARG targets (*n* = 15 total) ranged from 3 to 11 at any single farm, and from 0 to 8 in any single soil sample. The distribution of multi-gene detection at the farm, core, and sample level is displayed in (**Supplementary Figure S2**).

Each of the 98 cores was split into 0.0–7.6 cm and 7.6–15.2 cm depths. Although some of the soil physical, chemical, and biological properties differed with depth (**Table 2**), significant depth-based differences were generally not observed for individual resistance genes. The only ARG for which a difference was observed was *tet*(L). When the depth-based data was analyzed at the core level by farm, *tet*(L) was detected more frequently (*P* = 0.025) in the surface soils (0–7.1 cm) compared to soils from the lower depth (7.1–15.2 cm). Since major depth-based differences were not observed, further analysis of ARGs and soil parameters were performed only on the surface samples.

**Table 2:** Mean soil measurements by depth.

Soil factor	Mean 0.0–7.6 cm	Mean 7.6–15.2 cm	*P*-value
Coarse particulate organic matter (g/kg soil)	0.44	0.26	<0.0001
Fine particulate organic matter (g/kg soil)	0.60	0.61	-
Organic nitrogen (g/kg soil)	0.21	0.17	<0.0001
Organic carbon (g/kg soil)	2.02	1.67	-
Carbon (g/kg soil)	9.78	9.60	-
Large macroaggregates (% soil wt)	15.28	16.53	-
Small macroaggregates (% soil wt)	38.01	38.47	-
Micro aggregates (% soil wt)	23.90	24.92	-
Total water saturation (C/kg soil)	77.18	79.92	-
pH (unitless)	7.02	6.68	-
Buffer pH^∗^ (unitless)	7.09	7.02	-
Electrical conductivity (dS/m)	0.39	0.35	<0.05
Soil organic matter (%)	3.30	2.83	<0.0001
Nitrate [NO_3_] (mg/kg soil)	19.22	11.75	<0.05
Potassium [K] (mg/kg soil)	672.26	544.10	<0.05
Sulfur [S] (mg/kg)	14.18	12.87	<0.05
Zinc [Zn] (mg/kg soil)	3.14	2.16	<0.05
Calcium [Ca] (mg/kg soil)	3129.54	3117.73	-
Magnesium [Mg] (mg/kg soil)	384.36	387.46	-
Sodium [Na] (mg/kg soil)	18.48	24.54	-
Cation exchange capacity (cmol/kg)	21.33	21.50	-
Mehlich-3 phosphorus (mg/kg)	105.68	82.53	-
Total fatty acid (nmol/g soil)	112.15	70.85	<0.0001
Fatty acids fungi:bacteria (ratio)	0.30	0.22	<0.0001
Fatty acids bacteria (nmol/g soil)	56.59	37.29	<0.0001
Fatty acids actinomycetes (nmol/g soil)	7.35	4.34	<0.0001
Fatty acids cyclopropyl (nmol/g soil)	10.28	7.62	<0.0001
Fatty acids bacteria:cyclopropyl (ratio)	5.59	4.97	<0.0001
Fatty acids eukaryotes (nmol/g soil)	3.62	2.33	<0.0001
Fatty acids arbuscular mycorrhizal fungi [AMF] (nmol/g soil)	7.60	6.16	<0.05
Fatty acids saprophtes:fungi (ratio)	14.62	7.04	<0.0001
Sand (%)	19.70	20.01	-
Clay (%)	28.11	30.40	-
Silt (%)	50.37	48.10	-

*^∗^Buffer pH is used to determine the lime rate. A buffering solution of lime that is 0.6 effective Ca carbonate equivalent is added to each sample. To determine lime recommendation, pH is compared to buffer pH. If the difference is large, it suggests that the soil pH is easily changed.*

For each tetracycline resistance gene, mean values for soil parameters in the upper surface cores (0–7.1 cm) were compared for the samples that were positive vs negative for each ARG (**Table 3**). **Table 3** reports that 86 out of 476 gene-by-soil-parameter analyses were statistically significant. This analysis examined mean values for each soil parameter in the ARG positive samples, compared to the mean values in the ARG negative samples. Examining this set of data for those results likely to be biologically significant, four stand out because they were significant for four or more genes which trend positively for that soil parameter. EC, Ca, Na, and Mehlich-3 phosphorus (MehP) values were all consistently higher in the ARG positive soils. These four parameters are related to each other and together influence EC. In addition to having higher mean values for the positive ARG soils, these four measures (EC, Ca, Na, and MehP) were also positively correlated with the total number of ARG-positive targets (**Supplementary Table S2**). The relationships between number of detected resistance genes and soil physical and chemical parameters were examined using Pearson Correlation Coefficients (**Supplementary Table S2**). Significant differences (*P* < 0.05) or tendencies to differ (*P* < 0.1) were observed. The proportion of positive samples that were and were not exposed to manure within three years of collection are described in **Supplementary Table S3**, with statistically significant increases of *sulI*, *tet*(G), and *tet*(O) in the manured plots, and *tet*(D) in the non-manured plots.

**Table 3:** Mean soil values for ARG positive samples.

**Soil factor**	**Mean All^§^**	**Tetracycline resistance gene**														
	**(*n* = 98)**	**A**	**B**	**C**	**D**	**E**	**K**	**G**	**L**	**M**	**O**	**A(P)**	**Q**	**S**	**X**	***Sul*1**
Number of samples positive >		6	3	0	53	26	1	108	18	17	19	58	98	92	60	
Coarse particulate organic matter (g/kg soil)	0.44	-	-	-	-	-	-	-	-	-	-	0.33*	-	-	-	-
Fine particulate organic matter (g/kg soil)	0.60	-	-	-	-	-	-	0.53	0.42	0.45	-	0.51	-	-	-	-
Organic nitrogen (g/kg soil)	0.21	-	-	-	-	-	-	0.20	0.24*	-	0.26	-	0.22	-	-	-
Organic carbon (g/kg soil)	2.0	-	0.4	-	-	-	-	1.9*	-	-	2.5*	-	2.1	-	-	-
Carbon (g/kg soil)	9.7	-	4.15	-	-	-	-	-	9.4	9.3	9.4	10.1	9.9	-	-	9.4
Large macroaggregates (% soil wt)	15	-	-	-	-	-	-	-	-	-	-	-	-	12*	-	-
Small macroaggregates (% soil wt)	38	-	-	-	-	49	-	-	-	-	-	42	41	-	-	-
Micro aggregates (% soil wt)	24	-	-	-	-	-	-	-	-	-	-	-	-	-	27*	-
Total water saturation (C/kg soil)	77	-	49	-	83	85	-	-	-	70	-	-	80	-	-	-
pH (unitless)	7.0	-	8.3*	-	6.7	7.5	-	7.1	-	-	-	7.2*	-	-	-	-
Buffer pH (unitless)	7.0	-	-	-	-	-	-	7.1	-	-	6.9	7.2*	-	-	-	-
Electrical conductivity (dS/m)	0.39	-	0.61*	-	0.43	0.46	-	-	-	-	-	0.42*	-	-	-	-
Soil organic matter (%)	3.3	-	5.90	-	-	-	-	3.2*	-	-	3.9	-	3.5	-	-	-
Nitrate [NO_3_](mg/kg soil)	19	-	-	-	24*	30	-	-	-	-	-	-	-	-	-	-
Potassium [K] (mg/kg soil)	672	-	-	-	-	382	-	-	-	-	958	-	-	-	-	-
Sulfur [S] (mg/kg)	14	-	-	-	-	-	-	-	-	-	16	-	-	-	-	-
Zinc [Zn] (mg/kg soil)	3.1	-	-	-	-	-	-	2.5*	6.1	5.7	7.5	-	-	4.3	-	-
Calcium [Ca] (mg/kg soil)	3129	-	4694	-	-	4028	-	-	-	-	-	159	3309	-	-	-
Magnesium [Mg] (mg/kg soil)	384	657	998	-	-	300	-	-	521	481	530	-	-	-	-	-
Sodium [Na] (mg/kg soil)	18	-	302	-	-	-	-	-	38	45	55	-	-	24*	-	-
Cation exchange capacity (cmol/kg)	21	-	36	-	-	24	-	-	24	-	25	23	-	-	19	-
Mehlich-3 phosphorus (mg/kg)	105	-	484	-	-	-	-	-	229	228	263	-	-	140	-	153*
Total fatty acid (nmol/g soil)	112	-	239	-	-	-	-	-	-	133	146	-	-	-	-	-
Fatty acids fungi:bacteria (ratio)	0.30	-	-	-	-	-	-	-	-	-	-	-	-	-	0.35	-
Fatty acids bacteria (nmol/g soil)	56	-	109	-	-	-	-	-	65*	-	76	-	-	-	-	-
Fatty acids actinomycetes (nmol/g soil)	7.35	-	-	-	-	6.3*	-	6.2*	-	-	10	-	-	-	-	-
Fatty acids cyclopropyl (nmol/g soil)	10	-	27	-	-	-	-	-	5.4	-	15	-	-	-	-	-
Fatty acids bacteria:cyclopropyl (ratio)	5.6	-	4.0	-	5.4	-	-	-	-	-	5.1	-	-	-	5.8	5.8*
Fatty acids eukaryotes (nmol/g soil)	3.6	-	7.8	-	-	-	-	-	-	4.4	-	-	-	-	-	-
Fatty acids arbuscular mycorrhizal fungi (nmol/g soil)	7.6	-	45^∗^	-	-	-	-	-	-	11.2	11^∗^	-	-	-	-	-
Fatty acids saprophtes:fungi (ratio)	15	-	-	-	-	-	-	-	-	-	-	-	-	-	16	-
Sand (%)	20	-	-	-	15^∗^	27^∗^	-	-	12^∗^	-	-	-	-	14	-	-
Clay (%)	28	-	-	-	-	-	-	31^∗^	38^∗^	-	-	-	-	31^∗^	24^∗^	-
Silt (%)	50	-	-	-	-	40	-	-	-	-	-	-	-	-	-	-

*Listed values have a P ≤ 0.05, except where noted by an asterisk, denoting P ≤ 0.01. “–” indicates no significant difference from the mean for all (combined ARG positive and negative) samples. ^§^ The number of samples in the positive group represents all 98 samples, measured at both depths.*

Examining which gene targets had similar results for individual soil parameters (**Table 3**), EC and Ca had significantly higher mean values in samples where *tet*(B) and *tetA*(P) were detected. The Na and MehP values were higher in soil samples where *tet*(B), *tet*(L), *tet*(M), *tet*(O), and *tet*(S) were detected. Organic carbon, soil organic matter, and organic nitrogen measurements tended to have lower mean values in soils positive for *tet*(G) but were greater in soils positive for *tet*(B), *tet*(O), and *tet*(Q). The *tet*(B) gene appears at first glance to be most frequently associated with non-random changes in soil properties in positive compared to negative soils, however, note that there are only three positive samples in this group, so it is unlikely that there is any biological significance to these numbers (**Table 3**).

**FIGURE 2:**
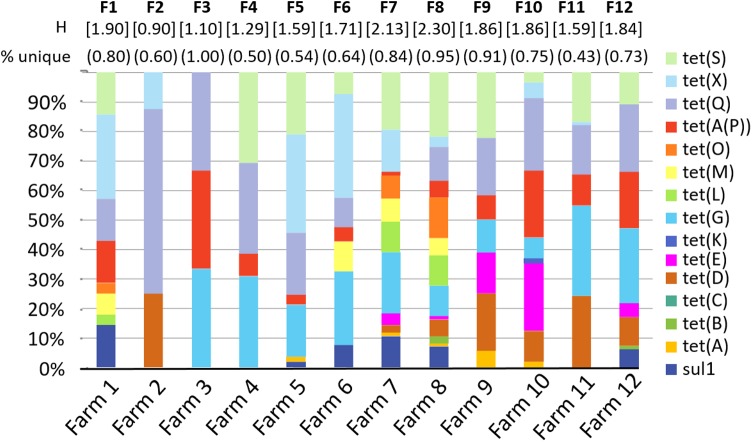
Diversity of selected antibiotic resistance genes by farm. Graph shows the percent of samples from each farm that were positive for each gene. “Percent unique” indicates the number of sample ARG profiles that are unique for each farm. For example, in farm 1, 8 of the 10 profiles were unique = 0.80. *H* is the Shannon diversity index for each farm. “F1–F12” indicates farms 1–12.

For each sample, each ARG is coded as detected = 1 or not detected = 0. These values are concatenated (i.e., linked together in a series) to create an ARG profile or fingerprint (Durso et al., 2011), serving as a molecular antibiogram. The ARG diversity profiles of the 12 farms sampled is presented in **Figure 2**. On average, 72% of the profiles were unique for each farm, with a range between 43% and 100% of profiles from each farm found exclusively in that farm With the exception of Farm 11, the majority of the samples within each farm had a unique ARG profile (range 0.43–1.00, mean 0.72, median 0.74), where a value of 1.0 indicates that every sample had a unique profile.

Using the SAS two sample test of equality of proportions (SAS Institute, 2008), we compared frequency of detection of targets from the current set of certified organic farm soils with results from a previously published set of native Nebraskan prairie soils (Durso et al., 2016). Significant differences were seen in the frequency of detection from certified organic farms compared to native prairie soils for 12 of 15 targets at the farm level (**Table 4**).

**Table 4:** Comparison of tetracycline and sulfonamide resistance gene prevalence in organic farms and native prairies in Nebraska.

Gene	Mechanism	Conventional manure prevalence (%)	Organic farm soil (*n* = 98)	Prairie soil (*n* = 100)	*P*-value
Sul1	Enzyme	100^A,B^	16	91	<0.0001
*tet*(A)	Efflux	8^C^	2	52	<0.0001
*tet*(B)	Efflux	0–4^B,C^	2	27	<0.0001
*tet*(C)	Efflux	0–100%^B,C,D^	0	14	0.0001
*tet*(D)	Efflux	No data	29	55	0.0004
*tet*(E)	Efflux	28^B^	14	15	0.887
*tet*(G)	Efflux	No data	56	15	<0.0001
*tet*(K)	Efflux	No data	0	9	0.0024
*tet*(L)	Efflux	No data	13	34	0.0006
*tet*(M)	Ribosomal	80–100%^B,D^	11	15	0.4316
*tet*(O)	Ribosomal	85–100%^B,D^	8	37	<0.0001
*tetA*(P)	Ribosomal	No data	30	17	0.359
*tet*(Q)	Ribosomal	80–100%^B,D^	48	0	<0.0001
*tet*(S)	Ribosomal	49^B^	45	12	<0.0001
*tet*(X)	Enzymatic	No data	33	2	<0.0001
Mean # ARGs^∗∗^			3.07	3.94	

P-value is for comparison of gene % positive in organic vs prairie soils. Manure prevalence % values are calculated from data from peer-reviewed publications that measured gene prevalence from various manure-impacted substrates. ^A^Data from Martietal. (2014). ^B^Data from Storteboometal. (2010). ^C^Data from Sengeløvetal. (2003). ^D^Data from Jindaletal. (2006). **Based on n = 15 assayed for this study.

## Discussion

Organic farms present a unique opportunity to determine impacts of agriculture on antibiotic resistance in soil, withoutction practices. Soils from 12 USDA certified organic farms in Nebraska were probed for the presence of tetracycline and sulfonamide resistance genes. All farms were positive for at least three, and up to 12 of the 15 assayed genes, demonstrating that ARGs are common in agricultural soils, even in the absence of routine antibiotic drug or pesticide use. These data support other work done in organic farming operations examining ARGs in organic cattle, swine, and poultry production (Stanton et al., 2011; Rothrock et al., 2016a; Sancheza et al., 2016), where ARGs were also detected even when antibiotic drugs were not administered to animals. It was not surprising to detect sulfonamide and/or tetracycline ARGs at every farm sampled, as they occur naturally in soils, and have been detected in soils and water from around the globe, including ungrazed native prairie soils from the same region of Nebraska in which this study was conducted (D’Costa et al., 2006, 2007; Allen et al., 2010; Durso et al., 2012, 2016; Cytryn, 2013).

There is broad consensus that agricultural antibiotic resistance needs to be reduced, but little information is available to inform what a target level should be, and no consensus on which targets to measure. As part of identifying which targets to measure in agricultural and environmental settings, it is informative to examine the frequency of detection for the tetracycline and sulfonamide gene targets in the 12 Nebraskan certified organic farms. In this instance, *tet*(G), *tet*(Q), *tet*(S), *tet*(X), and *tetA*(P) were most frequently detected (**Figure 1**), and are recommend as the most informative for future studies in these soils. The *sul*(I) gene has been proposed as a marker of human impacts (Pruden et al., 2006). In the current study *sul*(I) was detected at 50% of the farms, but in only 14% of the individual soil samples. This suggests that the utility of this gene as a general marker of anthropogenic agricultural activity might vary depending on the frequency and depth of sampling.

No statistically significant differences were observed for the incidence of various resistance genes from soil collected between 0 and 7.6 cm and that from 7.6 to 15.2 cm samples, with exception of *tet*(L). It is unclear from the data if the *tet*(L) result is biologically significant, as there were only three farms positive for *tet*(L) in this study, and the differences between the depths can be attributed to values from a single farm. Because the two depths compared in this study are both found within in the upper soil horizon, we conclude that these soils can be sampled within the top 15.2 cm without affecting ARG prevalence data. We know that bacterial phylogeny is correlated with ARG profiles (Fosberg et al., 2016), so it is expected that changes in a bacterial community structure will impact overall ARG carriage. However, for this set of soil and ARG targets, no changes in ARG profiles were observed at the two depths. This is an interesting disconnect with our current understanding that soil bacterial communities change with depth (Zhang et al., 2016), a finding that is reflected in the summary FAME data for these organic farms (**Table 2**). Although no qualitative differences in ARGs were observed for these soils, it may be that quantitative depth-based differences exist for the ARG targets in organic farm soils within the upper 15.2 cm, but they were not revealed with presence/absence data we collected. The ARG antibiogram results reported here are a strong indicator that additional sampling would likely yield additional unique profiles. It is possible, therefore, that the data reported here are an underestimation of the prevalence and distribution of the assayed genes.

### ARGs and Soil Properties

Antibiotic resistance genes are ubiquitous in soil (D’Costa et al., 2006; Durso et al., 2012), and the soil is thought to be a direct source for resistance genes that are associated with untreatable infectious disease in hospitals and clinics (Fosberg et al., 2012). As such, there is value in exploring the impact of soil properties on survival and persistence of ARGs in the soil matrix. It has been shown that the presence of metals in soil can provide a selective pressure for antibiotic resistance (Knapp et al., 2011), but little is known about the impacts of other physical and chemical parameters as they relate to antibiotic resistance. In this study, we identified relationships between multiple physical and chemical properties of the soil, and frequency of detection of sulfonamide and tetracycline resistance genes.

We observed higher EC values in ARG positive vs negative soils. EC is considered an indicator of soil health, influencing crop yield, nutrient availability and activity of soil microorganisms. EC values are also used to identify areas of manure deposition in feedlots and fields (Woodbury et al., 2009). Manure is a common amendment in organic systems, whether deposited via grazing or applied directly as a soil amendment, and it is known to enrich for ARGs in the soil (Udikovic-Kolic et al., 2014; Kyselkova et al., 2015), but this study was specifically not structured to discern the specific role of manures on ARGs in organic production systems. However, statistically significant greater numbers of ARGs were detected at sites having some history of manure application (**Supplementary Table S3**). If the patterns observed in this study apply more broadly, then EC measurements might be helpful in identifying soil regions that are more or less likely to be enriched for tetracycline or sulfonamide resistance genes. Soil Ca, Na, and MehP values were also consistently higher in the ARG positive soils, and may also be useful indicators either individually or as they related to and influence EC.

Sand, clay, TotWSA, pH, C, CEC, Bac:Cyclo, and FPOM did not seem to cluster with the other three groups or with each other, and they had varying relationships with tetracycline resistance genes. Interestingly FPOM had a consistently lower mean value with selected ARG targets [*tet*(G), *tet*(L), *tet*(M), *tet*(O), *tet*A(P)]. FPOM is an easily decomposable part of non-living soil organic matter. It provides resources for microorganisms and nutrients for plant growth. It is possible that the patterns we observed were related to complex interactions involved in active rhizosphere growth. Fatty acid data support the idea that there were active rhizosphere interactions in these soils. The cyclopropane fatty acids are found in a subset of Gram-negative bacteria, including a number of enteric and gut-associated bacteria like *Escherichia* and *Salmonella*, as well as soil dwelling bacteria such as *Rhizobium* (Grogan and Cronan, 1997). Because of the large number of enterics in this group, this fatty acid profile is of particular interest when exploring antibiotic resistance. We observed two ARG targets [*tet*(B), *tet*(O)] associated with significantly higher cyclopropane values as measured by fatty acid methyl ester analysis.

### Comparison With Pristine and Conventional Agriculture Sites

The Nebraskan certified organic farm data can be compared to previous data collected from 20 ungrazed native prairie sites, also in Nebraska (Durso et al., 2016). Identical methods were used for gene detection in both studies. Surprisingly, of the 12 targets that were significantly different between certified organic farm and prairie sites, 8 of 12 were less frequently detected in the farm soils than the prairie soils. We initially assumed that anthropogenic practices, such as farming, were likely to increase any measure of AR. However, in this instance we observed that the native prairies had “more resistance” than the farm soils, as measured by frequency of detection of selected ARG targets. Additionally, the mean number of different ARGs (*n* = 15 total) in the native prairie soils was 3.94, compared to only 3.07 for the organic farms. Again, numerically, the native prairie soils have “more resistance” than the farm soils. Since ARGs are, for the most part, carried inside of bacteria, and since bacterial phylogeny has a strong influence on the types of ARGs present in a sample (Fosberg et al., 2016), the fact that ARGs were more frequently detected in native prairie soils, and that there was a greater diversity of tetracycline resistance genes in native prairie soils, could potentially be explained by the expected greater microbial diversity in native prairie compared to farmed soils (Convention on Biological Diversity, 2010). Importantly, these data compare the number of different gene types, and do not take into account the absolute amount of each gene present. Our conclusions do not exclude the possibility that agricultural systems might have a greater total number of the target genes (absolute number or per 16S), as that was not measured as part of the current study. It is also important to note that there is currently no direct evidence that links soil ARG numbers or diversity with human health outcomes: the data collected in this study was not intended to address risk to human populations from agriculture. Finally, gene-based studies, such as the one reported here, can provide a valuable insight into the ecology of ARGs in agroecosystems, but PCR methods only reveal if a target is present in a sample. We have no information on whether or not the gene is expressed, or whether the gene is contained within a viable cell.

There are three main mechanisms of action for tetracycline resistance (**Table 4**). When the tetracycline resistance gene results were sorted by gene mechanism of action, the tetracycline efflux genes were generally present in higher frequency in the prairie soils, while the genes with ribosomal protection and enzymatic mechanisms of action were generally present in higher frequency in the organic farm soils. Individual ARGs each have their own ecologies (Durso et al., 2016). And although the current study design prevents us from drawing conclusions beyond the specific sites studied, the interpretation of our current results raises the possibility that there might be functional ecological significance that correlates with tetracycline resistance gene mechanism of action.

The long-term applied goal of studies of these types is to identify which ARG targets are the most relevant for agricultural production settings, and provide a starting point for identifying realistic targets for ARGs on farms and in fields. To that end, despite limited data, we can also compare our organic farm soil results to data collected from manures at conventional animal operations, where antibiotics would be used more frequently (**Table 4**). The *tet*(M) gene occurred at 15% or less of samples in both the organic farm and prairie soils. However, this same target was measured in 80–100% of conventionally raised animal manures in studies by Jindal et al. (2006) and Storteboom et al. (2010). This suggests that *tet*(M) prevalence could serve as a useful indicator of recent manure-borne resistance in the environment, and that there is potential utility in monitoring this gene over time when manures are land applied. Our conclusion on *tet*(M) supports European efforts that have identified *tet*(M) detection as a possible tool to track and monitor ARG transport from and within agricultural systems (Berendonk et al., 2015).

Organic farm soils can serve as a baseline for determining realistic target levels of ARGs in agricultural production settings. They also provide valuable information for studies probing the ecology of antibiotic resistance on farms and in fields. By comparing organic farms with less disturbed soils, such as native prairies, we can start to determine what kinds of impacts agricultural production practices may have on multiple measures of resistance. It is unclear if the relationships we observed are due to management, underlying macroecological (i.e., weather), or geophysical (i.e., soil type) factors. Additional studies are needed to determine if these relationships are broadly applicable across different spatial and temporal scales.

## Author Contributions

This work was conceived and planned by LD and MC, with input from DM, HW, and CW. Samples were collected and processed by RD and CW, and tested in the laboratory by MC, BC, and RD. Analysis was performed by MC, LD, DM, and BC, with help from HW, RD, and CW. The manuscript was drafted by MC and LD, with substantial input from DM, HW, BC, RD, and CW.

## Disclaimer

Mention of trade names or commercial products in this article is solely for the purpose of providing specific information and does not imply recommendation or endorsement by the U.S. Department of Agriculture. USDA is an equal opportunity provider and employer.

## Conflict of Interest Statement

The authors declare that the research was conducted in the absence of any commercial or financial relationships that could be construed as a potential conflict of interest.

## Supplementary Material

FIGURE S1 Percent of samples per farm with at least one of the targeted antibiotic resistance genes.

FIGURE S2 Number of target antibiotic resistance genes per sample **(A)** and farm **(B)**.

TABLE S1 PCR primers and annealing temperatures.

TABLE S2 Pearson correlation coefficients, based on number of positive ARG targets (*n* = 15 total ARG targets) per sample. *P* < 0.05 is considered significant. *P* < 0.1 is reported as a possible trend.

TABLE S3 Proportion of positive samples coming from sites that had received recent manure.
