# Supplementary figures and images for: Tetracycline and Sulfonamide Antibiotic Resistance Genes in Soils From Nebraska Organic Farming Operations
Source: Front Microbiol. 2018 Jun 28;9:1283. doi: 10.3389/fmicb.2018.01283 (PMC6033193; doi:10.3389/fmicb.2018.01283)

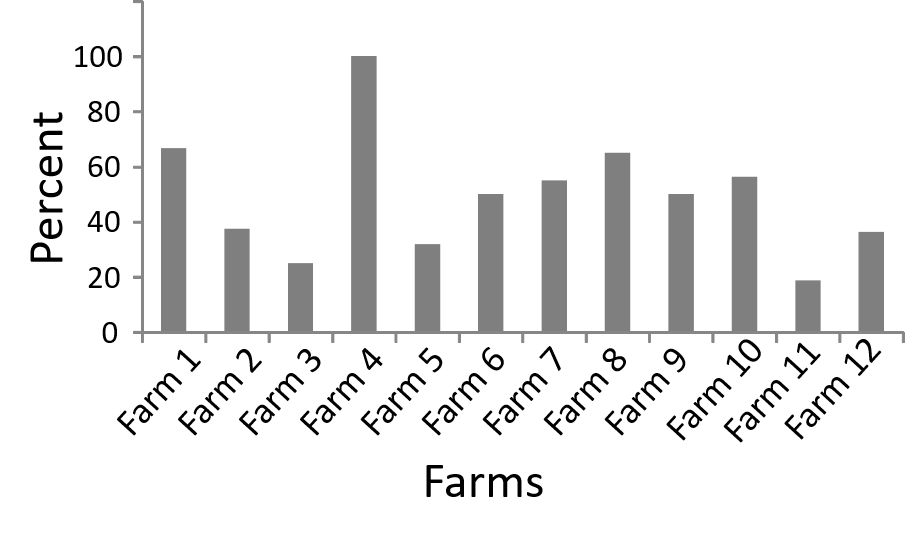

Supplement: FIGURE S1 — Percent of samples per farm with at least one of the targeted antibiotic resistance genes. [file Image_1.TIF]

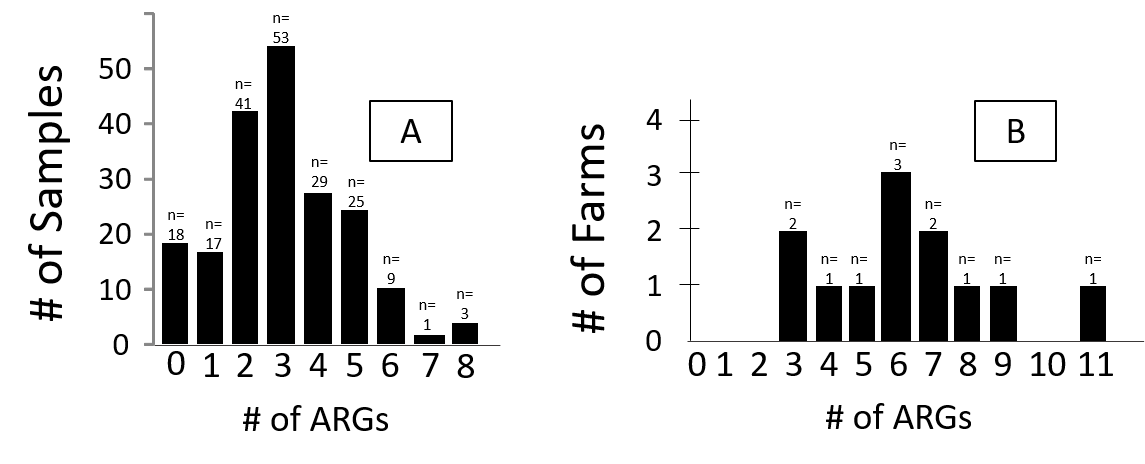

Supplement: FIGURE S2 — Number of target antibiotic resistance genes per sample (A) and farm (B). [file Image_2.TIF]
