# Supplementary material for: Tetracycline and Sulfonamide Antibiotic Resistance Genes in Soils From Nebraska Organic Farming Operations
Source: Front Microbiol. 2018 Jun 28;9:1283. doi: 10.3389/fmicb.2018.01283 (PMC6033193; doi:10.3389/fmicb.2018.01283)
Supplement: TABLE S1 — PCR primers and annealing temperatures. [file Data_Sheet_1.docx]

**Table S1.** PCR Primers and Annealing Temperatures

| Gene | Anneal  Temp | Primer Sequence | Size (bp) | Mechanism | Citation |
| --- | --- | --- | --- | --- | --- |
| *tet*(A) | 58ºC | GCTACATCCTGCTTGCCTTC  CATAGATCGCCGTGAAGAGG | 210 | Efflux | [Ng et al. 2001](http://www.sciencedirect.com/science/article/pii/S0890850801903639) |
| *tet*(B) | 58ºC | TTGGTTAGGGGCAAGTTTTG  GTAATGGGCCAATAACACCG | 659 | Efflux | [Ng et al. 2001](http://www.sciencedirect.com/science/article/pii/S0890850801903639) |
| *tet*(C) | 58ºC | CTTGAGAGCCTTCAACCCAG  ATGGTCGTCATCTACCTGCC | 418 | Efflux | [Ng et al. 2001](http://www.sciencedirect.com/science/article/pii/S0890850801903639) |
| *tet*(D) | 58ºC | AAACCATTACGGCATTCTGC  GACCGGATACACCATCCATC | 787 | Efflux | [Ng et al. 2001](http://www.sciencedirect.com/science/article/pii/S0890850801903639) |
| *tet*(E) | 58ºC | AAACCACATCCTCCATACGC  AAATAGGCCACAACCGTCAG | 278 | Efflux | [Ng et al. 2001](http://www.sciencedirect.com/science/article/pii/S0890850801903639) |
| *tet*(G) | 58ºC | CAGCTTTCGGATTCTTACGG  GATTGGTGAGGCTCGTTAGC | 844 | Efflux | [Ng et al. 2001](http://www.sciencedirect.com/science/article/pii/S0890850801903639) |
| *tet*(K) | 50ºC | TCGATAGGAACAGCAGTA  CAGCAGATCCTACTCCTT | 164 | Efflux | [Ng et al. 2001](http://www.sciencedirect.com/science/article/pii/S0890850801903639) |
| *tet*(L) | 57ºC | TCGTTAGCGTGCTGTCATTC  GTATCCCACCAATGTAGCCG | 267 | Efflux | [Ng et al. 2001](http://www.sciencedirect.com/science/article/pii/S0890850801903639) |
| *tet*(M) | 57ºC | GTGGACAAAGGTACAACGAG  CGGTAAAGTTCGTCACACAC | 406 | Ribosomal | [Ng et al. 2001](http://www.sciencedirect.com/science/article/pii/S0890850801903639) |
| *tet*(O) | 57ºC | AACTTAGGCATTCTGGCTCAC  TCCCACTGTTCCATATCGTCA | 515 | Ribosomal | [Ng et al. 2001](http://www.sciencedirect.com/science/article/pii/S0890850801903639) |
| *tetA*(P) | 58ºC | CTTGGATTGCGGAAGAAGAG  ATATGCCCATTTAACCACGC | 676 | Efflux | [Ng et al. 2001](http://www.sciencedirect.com/science/article/pii/S0890850801903639) |
| *tet*(Q) | 58ºC | TTATACTTCCTCCGGCATCG  ATCGGTTCGAGAATGTCCAC | 904 | Ribosomal | [Ng et al. 2001](http://www.sciencedirect.com/science/article/pii/S0890850801903639) |
| *tet*(S) | 58ºC | CATAGACAAGCCGTTGACC  ATGTTTTTGGAACGCCAGAG | 667 | Ribosomal | [Ng et al. 2001](http://www.sciencedirect.com/science/article/pii/S0890850801903639) |
| *tet*(X) | 58ºC | CAATAATTGGTGGTGGACCC  TTCTTACCTTGGACATCCCG | 468 | Enzymatic | [Ng et al. 2001](http://www.sciencedirect.com/science/article/pii/S0890850801903639) |
| *Sul* (I) | 64ºC | GACGAGATTGTGCGGTTCTT  GAGACCAATAGCGGAAGCC | 185 | Enzymatic | [Szczepanowski et al., 2009](https://pub.uni-bielefeld.de/publication/1591546) |

**Table S2.** Pearson Correlation Coefficients, based on number of positive ARG targets (n=15 total ARG targets) per sample. P<0.05 is considered significant. P<0.1 is reported as a possible trend.

|  | **Soil Factor** | **Correlation** | **P values** |
| --- | --- | --- | --- |
|  | **Coarse Particulate Organic Matter (*g/kg soil*)** | -- | -- |
|  | **Fine Particulate Organic Matter (*g/kg soil*)** | -0.30 | 0.003 |
|  | **Organic Nitrogen (*g ON/kg soil*)** | 0.17 | 0.090 |
|  | **Organic Carbon (*g OC/kg soil*)** | 0.19 | 0.063 |
|  | **Carbon (*g C/kg soil*)** | -- | -- |
|  | **Large macroaggregates (% soil wt)** | -0.20 | 0.043 |
|  | **Small macroaggregates (% soil wt)** | 0.26 | 0.010 |
|  | **Micro Aggregates (% soil wt)** | -- | -- |
|  | **Total Water Saturation (*C/kg soil*)** | -- | -- |
|  | **pH (*unitless*)** | -- | -- |
|  | **Buffer (*unitless*)** | -- | -- |
|  | **Electrical Conductivity (*dS/m*)** | 0.22 | 0.027 |
|  | **Soil Organic Matter (*percent*)** | -- | -- |
|  | **Nitrate [NO_3_](*mg N/kg soil*)** | -- | -- |
|  | **Potassium [K] (*mg K/kg soil*)** | -- | -- |
|  | **Sulfur [S] (*mg/kg*)** | -- | -- |
|  | **Zinc [Zn] (*Zn mg/kg soil*)** | 0.19 | 0.056 |
|  | **Calcium [Ca] (*mg Ca/kg soil*)** | 0.24 | 0.017 |
|  | **Magnesium [Mg] (*mg Mg/kg soil*)** | -- | -- |
|  | **Sodium [Na] (*mg Na/kg soil*)** | 0.24 | 0.016 |
|  | **Cation Exchange Capacity (*cmol/kg*)** | 0.25 | 0.012 |
|  | **Mehlic-3 Phosphorus (*mg/kg*)** | 0.28 | 0.006 |
|  | **Total Fatty Acid (*nmol/g soil*)** | -- | -- |
|  | **Fatty Acids Fungi:Bacteria (*ratio*)** | -- | -- |
|  | **Fatty Acids Bacteria (*nmol/g soil*)** | -- | -- |
|  | **Fatty Acids Actinomycetes (*nmol/g soil*)** | -- | -- |
|  | **Fatty Acids Cyclopropyl (*nmol/g soil*)** | 0.18 | 0.073 |
|  | **Fatty Acids Bacteria:Cyclopropyl (*ratio*)** | -- | -- |
|  | **Fatty Acids Eukaryotes (*nmol/g soil*)** | -- | -- |
|  | **Fatty Acids Arbuscular Mycorrhizal Fungi [AMF] (*nmol/g soil*)** | -- | -- |
|  | **Fatty Acids Saprophtes:Fungi (*ratio*)** | -- | -- |
|  | **Sand (*Percent*)** | -- | -- |
|  | **Clay (*Percent*)** | -- | -- |
|  | **Silt (*Percent*)** | -- | -- |

**Table S3.** Proportion of positive samples coming from sites that had received recent manure.

| **ARG** | **No Manure** | **Recent Manure** | **P Diff** |
| --- | --- | --- | --- |
| *sul*1 | 0.033 | 0.221 | 0.021 |
| *tet(*A) | 0.033 | 0.015 | 0.548 |
| *tet(*B) | 0.00 | 0.015 | 0.540 |
| *tet(*C) | -- | -- | -- |
| *tet(*D) | 0.433 | 0.235 | 0.048 |
| *tet(*E) | 0.100 | 0.162 | 0.421 |
| *tet(*G) | 0.400 | 0.632 | 0.033 |
| *tet(*K) | -- | -- | -- |
| *tet(*L) | 0.033 | 0.176 | 0.054 |
| *tet(*M) | 0.033 | 0.147 | 0.100 |
| *tet(*O) | 0.00 | 0.118 | 0.050 |
| *tetA*(P) | 0.233 | 0.323 | 0.367 |
| *tet(*Q) | 0.400 | 0.515 | 0.295 |
| *tet(*S) | 0.400 | 0.471 | 0.517 |
| *tet(*X) | 0.333 | 0.323 | 0.924 |
